# Physiological ripples during sleep in scalp electroencephalogram of healthy infants

**DOI:** 10.1093/sleep/zsad247

**Published:** 2023-10-10

**Authors:** Kavyakantha Remakanthakurup Sindhu, Christopher Phan, Sara Anis, Aliza Riba, Cristal Garner, Amber L Magers, Nhi Tran, Amy L Maser, Katharine C Simon, Sara C Mednick, Daniel W Shrey, Beth A Lopour

**Affiliations:** Department of Biomedical Engineering, University of California, Irvine, Irvine, CA, USA; Department of Biomedical Engineering, University of California, Irvine, Irvine, CA, USA; Department of Biomedical Engineering, University of California, Irvine, Irvine, CA, USA; Division of Neurology, Children’s Hospital of Orange County, Orange, CA, USA; Division of Neurology, Children’s Hospital of Orange County, Orange, CA, USA; Division of Neurology, Children’s Hospital of Orange County, Orange, CA, USA; Division of Neurology, Children’s Hospital of Orange County, Orange, CA, USA; Division of Neurology, Children’s Hospital of Orange County, Orange, CA, USA; Department of Cognitive Sciences, University of California, Irvine, Irvine, CA, USA; Department of Cognitive Sciences, University of California, Irvine, Irvine, CA, USA; Division of Neurology, Children’s Hospital of Orange County, Orange, CA, USA; Department of Pediatrics, University of California, Irvine, Irvine, CA, USA; Department of Biomedical Engineering, University of California, Irvine, Irvine, CA, USA

## Introduction

During sleep, physiological ripple oscillations (80–250 Hz) occur throughout the brain [1, 2] and are thought to be significant markers of cognition [3]. While most studies of physiological ripples have relied on intracranial EEG recordings from patients with epilepsy, there is recent evidence that they can be recorded noninvasively using scalp EEG [4–8]. However, these events are infrequent and have low signal-to-noise ratios. Thus, some previous studies relied on manual event marking to maximize specificity, although it is time-consuming and introduces bias. Other studies utilized automated detection; however, they analyzed only brief EEG epochs. Moreover, several studies included participants with epilepsy, which confounds the measurement of physiological ripples, and used daytime EEG recordings following sleep deprivation, which alters sleep patterns and likely also ripple occurrence.

Therefore, we aimed to detect physiological ripples in an exclusively healthy population and obtain robust values of their spatiotemporal characteristics while addressing these limitations. We analyzed long-term scalp EEG recordings from healthy infants using an automated ripple detector, allowing us to incorporate multiple hours of data per participant. Studying infants also maximized the recordings’ signal-to-noise ratios, as their skulls are thinner than adults.

## Methods

This prospective study was approved by the Institutional Review Board of Children’s Hospital of Orange County (CHOC). Data were collected at CHOC from participants undergoing EEG monitoring to evaluate for infantile spasms, and those not exhibiting spasms were selected for analysis (Supplementary Appendix 1). Continuous overnight EEG was recorded at a 5000 Hz sampling rate using 19 electrodes placed according to the international 10–20 system. Segments of quiet and active sleep (in participants younger than 4 months) and N1, N2, N3, and REM sleep (in participants older than 4 months) were identified by registered polysomnographic technologists, extracted, and analyzed.

The data were first re-referenced to a longitudinal bipolar montage and then bandpass filtered from 80 to 250 Hz. Ripples were automatically detected using a previously validated algorithm [9–11] in 5-second intervals to minimize false detections due to muscle artifacts. The algorithm’s alpha parameter was optimized for each participant using a randomly selected 10-minute data segment.

A five-step automated artifact rejection algorithm (Supplementary Appendix 2) was used to exclude false detections. This included the following event feature cutoffs: (1) 50 μV maximum difference between consecutive raw data points, (2) maximum line length of 2000 $\sqrt{\mu V^{2}+s^{2}}$in the raw data, (3) maximum of 20 zero crossings in the raw data, (4) 20 μV maximum amplitude in the ripple band, and (5) 200 milliseconds maximum duration. These limits were based on previously reported ranges of these features [4, 12–14] or values obtained from representative 10-minute segments. MATLAB code for automated ripple detection and artifact rejection is included as Supplementary Material.

All events satisfying these criteria underwent visual validation by two independent reviewers. For the visually validated events in each channel, we calculated the ripple density, amplitude, duration, and peak frequency for each sleep stage and participant. Global ripple density was calculated by summing the densities of non-overlapping ripples across all electrodes. All statistical comparisons used the Wilcoxon rank sum test with Bonferroni correction.

## Results

**Figure 1.**
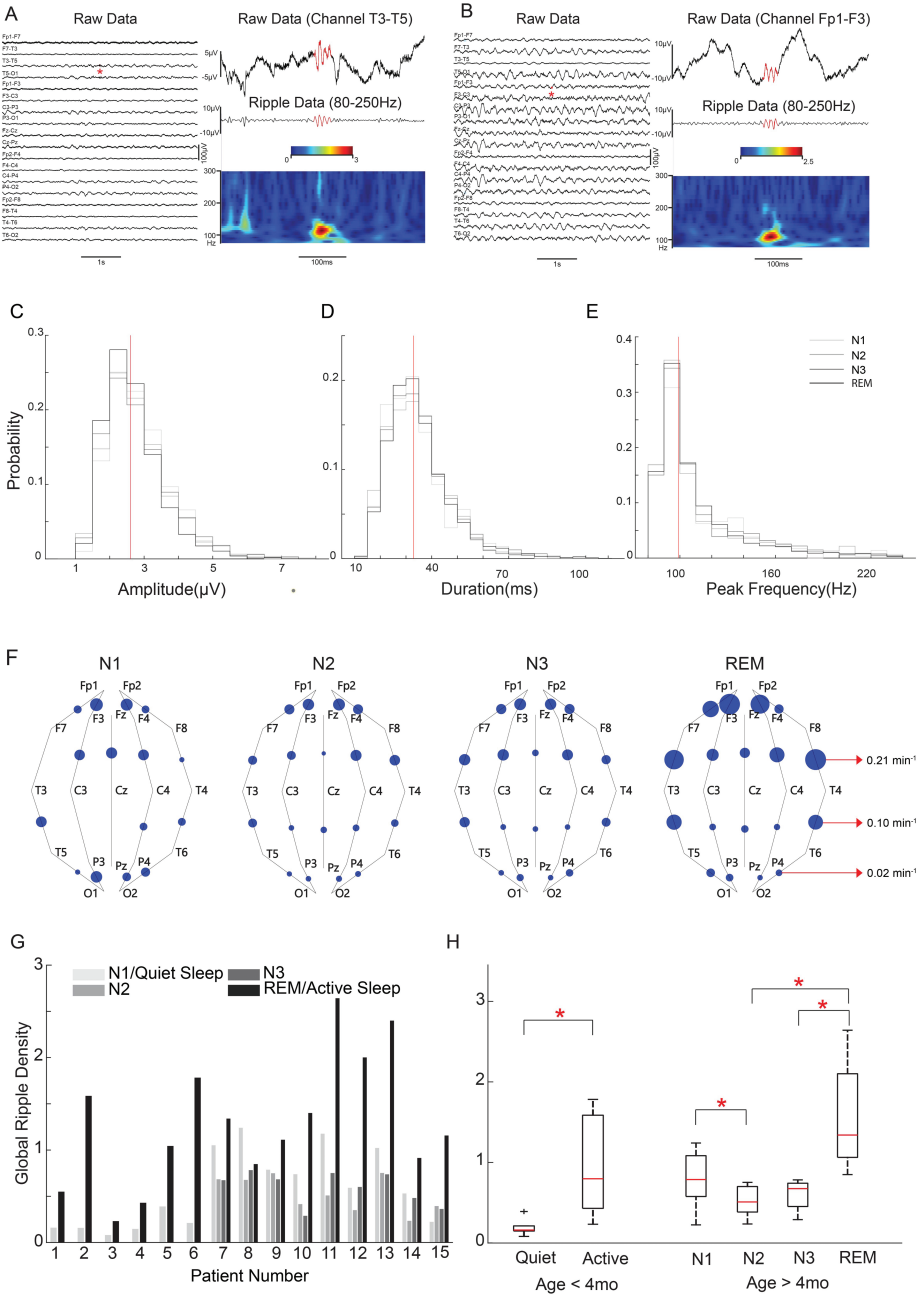
(A, B) Examples of ripples detected in the scalp EEG that passed visual validation. Histograms of (C) ripple amplitude, (D) ripple duration, and (E) ripple peak frequency. Vertical lines indicate the median across all sleep stages. (F) Spatial distribution of ripple densities for participants >4 months old. The area of each circle is proportional to the density for that bipolar channel pair. (G) Global ripple densities per sleep stage for each participant. Participants are listed in order by increasing age. (H) Boxplots of global ripple densities across all participants <4 months old (left) and > 4 months old (right). The boxplots were generated using data points corresponding to the average value for each participant. (* indicates *P*-value < 0.05, all *P*-values are corrected for multiple comparisons using the Bonferroni method).

Fifteen participants (median age: 6.3 months, IQR: 2.9–8.4 months) were included in this study, and a total of 184 hours of sleep EEG were obtained. Detailed patient information is given in Supplementary Table S1. The automatic detection and artifact rejection yielded 51 000 ripples across all participants, of which 11 718 (22.97%) were marked as true ripples through two-reviewer visual validation (Figure 1A, B).

The statistics for ripple features and densities are reported in Table 1 (Figures 1C-E). The highest global density, calculated by summing the number of ripples across all 19 channels, was 1.66 events per minute; therefore, in any 1 minute of data, most channels did not exhibit any ripples. We found lower amplitudes in REM than in NREM sleep (*p* < 0.001, Supplementary Figure S1A), but the effect size was small (Cohen’s *d* = 0.18). There were no significant differences in duration or peak frequency between REM and NREM stages (*p* > 0.5, Supplementary Figure S1B, C).

**Table 1.** Statistics for Ripple Characteristics

Participants younger than 4 months			
Feature	Median	25thpercentile	75th percentile
Amplitude (µV)	2.12	1.76	2.61
Duration (ms)	32.2	26.0	38.2
Peak Frequency (Hz)	99 Hz	92 Hz	115 Hz
Density—Frontal (min^−1^)	0.02	0.01	0.09
Density—Temporal (min^−1^)	0.06	0.02	0.08
Density—Prefrontal (min^−1^)	0.04	0.02	0.08
Density—Central (min^−1^)	0.02	0	0.03
Density—Parietal (min^−1^)	0.01	0	0.01
Density—Occipital (min^−1^)	0.01	0	0.01
Density—Anterior (min^−1^)	0.04	0.01	0.08
Density—Posterior (min^−1^)	0.03	0.02	0.03
Density—Left (min^−1^)	0.04	0.02	0.06
Density—Right (min^−1^)	0.03	0.02	0.05
**Participants older than 4 months**			
Amplitude (µV)	2.6	2.12	3.22
Duration (ms)	33.0	26.2	40.6
Peak Frequency (Hz)	99	92	118
Density—Frontal (min^−1^)	0.09	0.06	0.13
Density—Temporal (min^−1^)	0.07	0.06	0.09
Density—Prefrontal (min^−1^)	0.09	0.04	0.11
Density—Central (min^−1^)	0.02	0.02	0.06
Density—Parietal (min^−1^)	0.02	0.01	0.03
Density—Occipital (min^−1^)	0.04	0.02	0.05
Density—Anterior (min^−1^)	0.09	0.05	0.1
Density—Posterior (min^−1^)	0.04	0.03	0.05
Density—Left (min^−1^)	0.06	0.04	0.08
Density—Right (min^−1^)	0.06	0.05	0.07

The ripple density had a non-uniform spatial distribution that varied by sleep stage, for both age groups (Figures 1F, Supplementary Figure S2A). For participants younger than 4 months, ripple densities were not significantly different between brain regions (Supplementary Figure S2B). For participants older than 4 months, the highest densities were in frontal, temporal, and frontopolar regions across all sleep stages, and the densities in anterior regions were significantly higher than in posterior regions (Supplementary Figure S2C). Left and right hemispheric densities were not significantly different for either age group (Supplementary Figure S2B, C).

For participants younger than 4 months, the global ripple density in quiet sleep was significantly lower than in active sleep. This relationship was robust in individual participants and when assessed groupwise (Figure 1G, H). For participants older than 4 months, individuals generally showed the highest densities in REM sleep, followed by N1 sleep (Figure 1G). This was consistent with groupwise results (Figure 1H), in which REM sleep had significantly higher densities than N2 and N3, and N1 sleep densities were significantly higher than those in N2.

Lastly, we found that ripple amplitude and duration were correlated with participant age, while density and peak frequency were not (Supplementary Figure S6).

## Discussion

This is the most comprehensive study of scalp ripples in healthy human participants to date. It is the first to identify and characterize scalp ripples in long-term EEG (hours of data per participant, as opposed to minutes) while studying an exclusively healthy population. It is also the first to focus solely on infants and to include REM sleep in the analysis.

This study shows that physiological ripple density is the highest in REM sleep, contrary to what has been seen with epilepsy-associated, pathological ripples [15–17]. This may be because the two types of events are generated by different underlying mechanisms. Also, epileptic activity is suppressed in REM sleep [18], which could account for the low ripple densities observed in REM sleep in patients with epilepsy. Because most studies of physiological ripples in adults have excluded REM sleep, it is unclear if our finding of increased densities in REM sleep is specific to infants.

While muscle artifacts share characteristics with ripples, it is unlikely that this caused the high ripple densities seen during REM. In REM sleep, muscle activity is suppressed and is predominant in temporal and central regions, in contrast to the frontally predominant ripple patterns. Moreover, we found no significant differences in ripple duration or peak frequency when comparing REM to other sleep stages (Supplementary Figure S1, S3–S5). Lastly, ripple amplitude was slightly lower in REM compared to NREM sleep; the reverse would be expected if high amplitude muscle artifacts during REM triggered false detections. Similarly, eye movements during REM were unlikely to result in false ripple detections, as they are known to predominantly increase delta-band power, with minimal impact on higher frequencies.

A limitation of this study was its small sample size, owing to its prospective nature and the focus on infantile spasms, which is a rare disease. We analyzed large durations of sleep to mitigate this, but a larger cohort is needed to accurately quantify group variability. Furthermore, incorporating a wider age range would provide insights into age-based variation. This study is a first step towards understanding the significance of ripples during REM sleep for early brain development.

## Supplementary Material

zsad247_suppl_Supplementary_Material
