# Supplementary material for: Physiological ripples during sleep in scalp electroencephalogram of healthy infants
Source: Sleep. 2023 Oct 10;46(12):zsad247. doi: 10.1093/sleep/zsad247 (PMC10710989; doi:10.1093/sleep/zsad247)
Supplement: zsad247_suppl_Supplementary_Material [file zsad247_suppl_supplementary_material.docx]

­Supplementary Material

**Physiological ripples during sleep in scalp electroencephalogram of healthy infants**

Kavyakantha Remakanthakurup Sindhu^1^, Christopher Phan^1^, Sara Anis^1^, Aliza Riba^2^, Cristal Garner^2^, Amber L. Magers^2^, Nhi Tran^2^, Amy L. Maser^2^, Katharine C. Simon^3^, Sara C. Mednick^3^, Daniel W. Shrey^2,4^, Beth A. Lopour^1^

^1^ Department of Biomedical Engineering, University of California, Irvine, Irvine, CA, USA

^2^ Division of Neurology, Children’s Hospital of Orange County, Orange, CA, USA

^3^ Department of Cognitive Sciences, University of California, Irvine, Irvine, CA, USA

^4^ Department of Pediatrics, University of California, Irvine, Irvine, CA, USA

**Corresponding author:**

Beth Lopour

Department of Biomedical Engineering

3120 Natural Sciences II

Irvine, CA 92697-2715

[beth.lopour@uci.edu](mailto:beth.lopour@uci.edu)

***Table S1****: Subject demographics and EEG data characteristics. (QS – Quiet Sleep, AS – Active sleep)*

| **Subject No.** | **Sex/Age (Months)** | **N1/QS Duration (Hrs)** | **N2 Duration (Hrs)** | **N3 Duration (Hrs)** | **REM/AS Duration (Hrs)** | **Detection Threshold (α)** | **N1/QS Global HFO Density (per minute)** | **N2 Global HFO Density (per minute)** | **N3 Global HFO Density (per minute)** | **REM/AS Global HFO Density (per minute)** |
| --- | --- | --- | --- | --- | --- | --- | --- | --- | --- | --- |
| 1 | F/1.97 | 5.81 | N/A | N/A | 9.71 | 0.0025 | 0.16 | N/A | N/A | 0.55 |
| 2 | M/2.07 | 6.22 | N/A | N/A | 6.12 | 0.005 | 0.16 | N/A | N/A | 1.59 |
| 3 | F/2.2 | 4.35 | N/A | N/A | 6.12 | 0.001 | 0.08 | N/A | N/A | 0.23 |
| 4 | F/2.56 | 4.30 | N/A | N/A | 4.85 | 0.0025 | 0.15 | N/A | N/A | 0.43 |
| 5 | F/3.78 | 5.39 | N/A | N/A | 7.26 | 0.0025 | 0.39 | N/A | N/A | 1.04 |
| 6 | F/4.01 | 4.58 | N/A | N/A | 4.72 | 0.0025 | 0.21 | N/A | N/A | 1.78 |
| 7 | M/5.98 | 0.60 | 4.54 | 3.76 | 5.06 | 0.0025 | 1.05 | 0.68 | 0.67 | 1.34 |
| 8 | M/6.34 | 0.52 | 2.19 | 2.83 | 5.88 | 0.001 | 1.24 | 0.68 | 0.78 | 0.85 |
| 9 | F/6.96 | 1.44 | 9.95 | 6.37 | 12.58 | 0.0025 | 0.79 | 0.75 | 0.68 | 1.11 |
| 10 | F/7.42 | 0.56 | 4.09 | 3.41 | 3.84 | 0.005 | 0.74 | 0.42 | 0.29 | 1.40 |
| 11 | M/7.72 | 0.14 | 3.28 | 1.35 | 1.37 | 0.001 | 1.18 | 0.51 | 0.75 | 2.64 |
| 12 | F/8.57 | 0.42 | 5.00 | 2.44 | 3.82 | 0.001 | 0.59 | 0.35 | 0.6 | 2.00 |
| 13 | F/9.33 | 0.59 | 4.65 | 3.25 | 5.06 | 0.005 | 1.02 | 0.75 | 0.74 | 2.40 |
| 14 | F/11.14 | 0.31 | 3.27 | 2.73 | 1.80 | 0.0025 | 0.53 | 0.23 | 0.48 | 0.91 |
| 15 | F/11.27 | 0.30 | 2.67 | 2.71 | 1.94 | 0.001 | 0.22 | 0.39 | 0.36 | 1.16 |


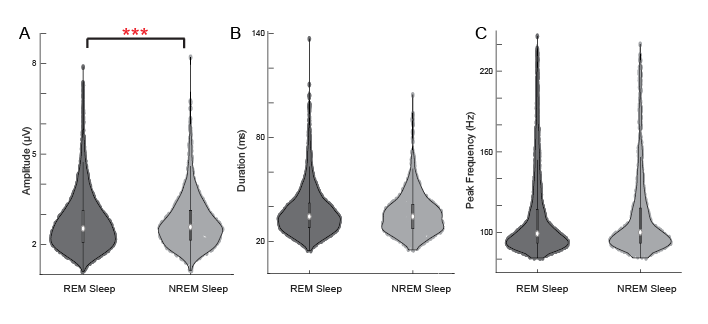
***Figure S1****. Comparison of ripple characteristics between REM and NREM sleep.* ***(A)*** *Amplitude* ***(B)*** *Duration, and* ***(C)*** *Peak frequency*


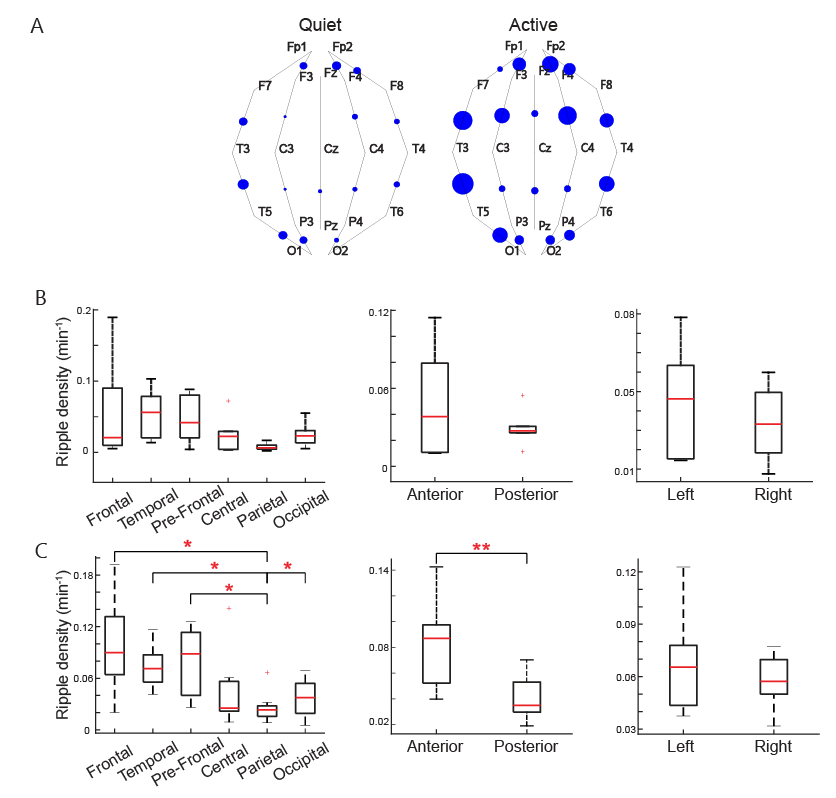


***Figure S2****.* ***(A)*** *Spatial distribution of ripple densities for subjects <4 months old.* ***(B)*** *Boxplots of mean (per subject) ripple densities for subjects <4 months old and* ***(C)*** *subjects >4 months old from different anatomical regions (left), anterior and posterior regions (middle), and left and right hemispheres (right). Regions are defined as frontal (Fp1-F3, Fp1-F7, Fp2-F4, Fp2-F8), temporal (F7-T3, T3-T5, F8-T4, T4-T6), prefrontal (F3-C3, F4-C4), central (Fz-Cz, Cz-Pz), parietal (C3-P3, C4-P4), and occipital (T3-O1, P3-O1, T4-O2, P4-O2). ( * indicates p-value < 0.05, ** indicates p-value < 0.01; all p-values are corrected for multiple comparisons using the Bonferroni method)*


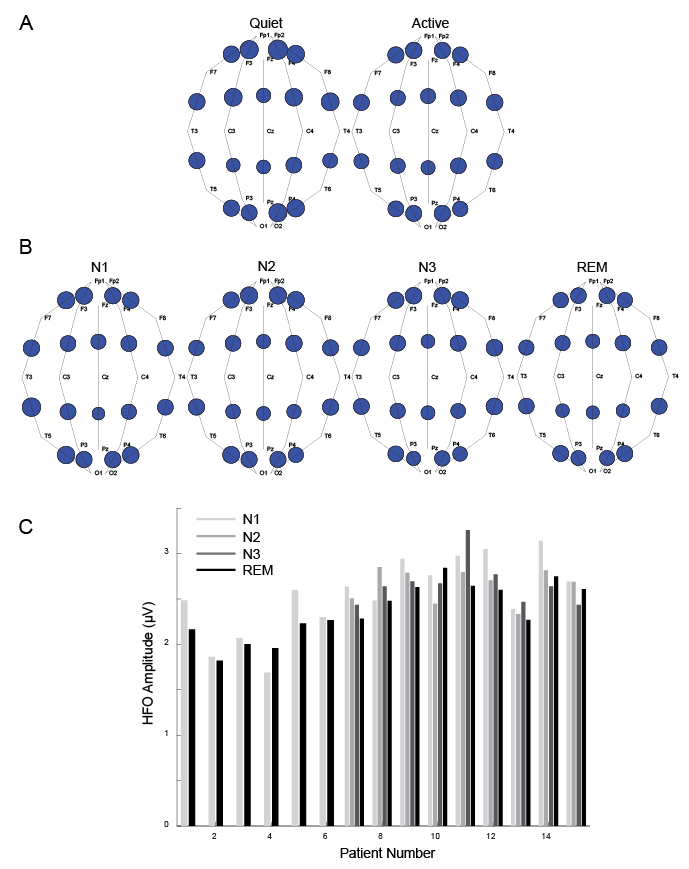


***Figure S3****. Spatial distribution of ripple amplitudes for* ***(A)*** *subjects <4 months old and* ***(B)*** *subjects >4 months old. The area of each blue circle is proportional to the amplitude for that bipolar channel pair. It can be seen that ripple amplitude is consistent across sleep stages and spatial locations.* ***(C)*** *Average amplitude of ripples per sleep stage across subjects*.

C


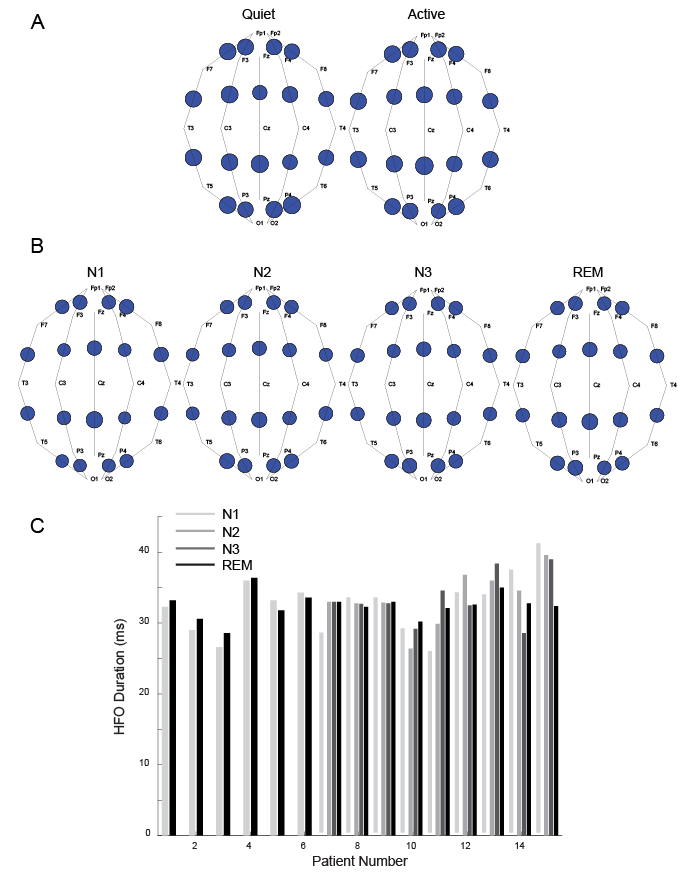


***Figure S4****. Spatial distribution of ripple durations for* ***(A)*** *subjects <4 months old and* ***(B)*** *subjects >4 months old. The area of each blue circle is proportional to the duration for that bipolar channel pair. It can be seen that ripple duration is consistent across sleep stages and spatial locations.* ***(C)*** *Average duration of ripples per sleep stage across subjects.*


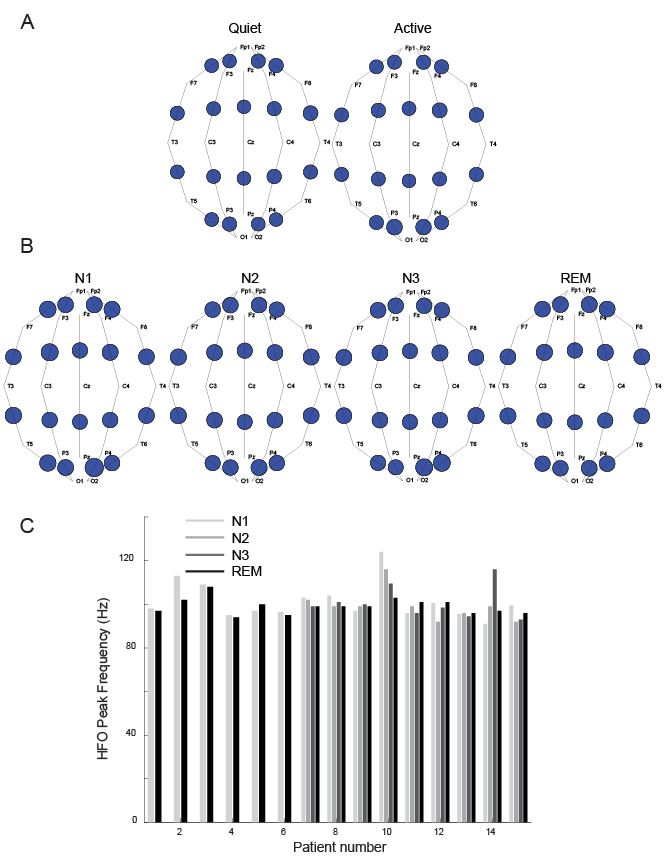


***Figure S5****. Spatial distribution of ripple peak frequencies for* ***(A)*** *subjects <4 months old and* ***(B)*** *subjects >4 months old. The area of each blue circle is proportional to the density for that bipolar channel pair. It can be seen that ripple peak frequency is consistent across sleep stages and spatial locations.* ***(C)*** *Average peak frequency of ripples per sleep stage across subjects.*


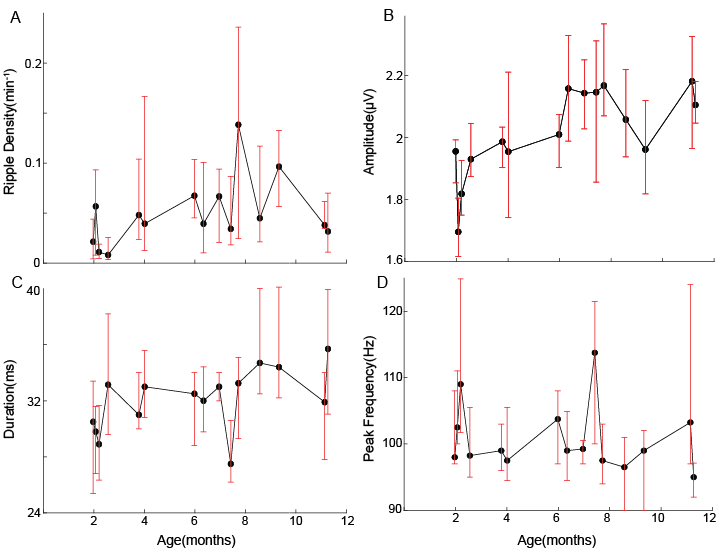


***Figure S6****.* *Ripple characteristics as a function of subject age:* ***(A)*** *Global ripple density,* ***(B)*** *ripple amplitude,* ***(C)*** *ripple duration, and* ***(D)*** *ripple peak frequency. In each subfigure, the median value across all channels is indicated by a filled black circle and the IQR is indicated by a red line.*

**Appendix-1: Subject Inclusion Criteria**

Subjects were undergoing inpatient EEG monitoring for suspicion of IS at CHOC between June 2017 and January 2019. To study physiological ripples, we selected for analysis all subjects that did not exhibit IS during monitoring. Further inclusion criteria included a normal Vineland developmental score (Sparrow et al., 2016) and normal MRI. Subjects were excluded if any of the following were true: seizures were observed, subject was on medication at any point in the study, subject had other medical diagnoses, technical error occurred during research recording, or initial results were suspicious for spasms. These exclusion criteria were applied to ensure the cohort consisted only of healthy infants.

**Appendix-2: Automatic Artifact Rejection**

Artifacts like muscle noise, DC shifts, and sharp events occur commonly in scalp EEG. These events can pass the threshold for automatic detection and be falsely detected as ripples. To reject these artifactual events, we placed limits on the duration, amplitude, maximum difference, line-length, and number of zero crossings in the detected event. In all cases, we chose a conservative threshold, erring on the side of keeping too many events rather than rejecting too many events, as we later visually validated each one.

1. **Maximum Duration**

Ripples in scalp EEG typically have a duration of 40-100 ms. Longer events tend to be associated with muscle noise. Therefore, events longer than 200ms in duration were rejected as artifacts.

1. **Maximum Amplitude**

Previous studies indicate that the RMS amplitude of scalp ripples lie in the range of 0.95-5.24 μV. Amplitudes that are much higher than this range were observed to be associated with events of non-neural origin, such as those created due to the filtering of sharp artifacts or spikes. Therefore, events with maximum amplitudes in the ripple band greater than 20 μV were rejected as artifacts.

1. **Maximum Difference**

When filtered in the ripple band, events like DC-shifts and fast transients appear similar to real ripples and hence are often detected by automatic algorithms. Such events are characterized by large fluctuations in amplitude in the raw data. Thus, candidate events with a maximum difference >50 µV between successive data points in the raw data were excluded.

1. **Line Length**

Muscle activity is a common source of scalp EEG artifacts. When filtered in the ripple band, these artifacts may look similar to a true ripple. However, they differ in that they often have a high amplitude in the raw data and a longer duration. To measure this, line length was calculated as the sum of distances between successive points in time for windows of EEG that included +/- 150 ms surrounding the candidate events. This accounts for the fact that false ripples due to muscle activity will likely be embedded in longer periods of high amplitude, high frequency activity. Candidate events occurring in windows with line length > 2000 $\sqrt{{\mu V}^{2}+s^{2}}$ in the raw data were excluded.

1. **Number of Zero Crossings**

Segments of raw data that excessively cross the zero line are considered artifactual and can be a source of false positive ripple detection; this is in contrast to a typical ripple, which rides on top of the lower frequency EEG activity, away from the zero line. Thus, candidate events with >20 zero crossings in the raw data were excluded. The number of zero crossings was calculated using the duration of the candidate event only.
